# Is the UN receiving ethical approval for its research with human participants?

**DOI:** 10.1136/jme-2023-109146

**Published:** 2024-02-27

**Authors:** Robert James Torrance, Maru Mormina, Sadath Sayeed, Anthony Kessel, Chang Ho Yoon, Beniamino Cislaghi

**Affiliations:** 1 London School of Hygiene and Tropical Medicine Faculty of Public Health and Policy, London, UK; 2 Faculty of Medical and Health Sciences, University of Auckland, Auckland, New Zealand; 3 African Centre of Epistemology and Philosophy of Science, University of Johannesburg, Johannesburg, South Africa; 4 David Geffen School of Medicine, University of California, Los Angeles, Los Angeles, California, USA; 5 University of Oxford Big Data Institute, Oxford, UK

**Keywords:** Ethics, Ethics- Research, Ethics Committees, Policy

## Abstract

This paper examines the institutional mechanisms supporting the ethical oversight of human participant research conducted by the United Nations (UN). The UN has served an instrumental role in shaping international standards on research ethics, which invariably require ethical oversight of all research studies with human participants. The authors’ experiences of conducting research collaboratively with UN agencies, in contrast, have led to concern that the UN frequently sponsors, or participates in, studies with human participants that have not received appropriate ethical oversight. It is argued that the institutional mechanisms in place to prevent research with human participants from being undertaken by the UN without ethical oversight do not, at present, extend substantively beyond the provision of guidelines and online training offered by a minority of UN bodies. The WHO and UNICEF are identified as notable exceptions, having implemented various measures to prevent health research with human participants from being undertaken without ethical oversight. Yet, it is highlighted that the WHO and UNICEF are not the only UN bodies that undertake health research with human participants and there are countless actors under the umbrella of the UN system that are regularly involved in non-health research with human participants. Arguments for the pursuit of the highest standard of ethical oversight by UN bodies are presented. Moving forward, the paper asks the question: is it time for the UN to set the standards for the oversight of ethical oversight?

## Introduction

 Hussein and Elmusharaf conducted a systematic review of human participant research undertaken during the armed conflict in Darfur (2004–2012) and found that only 1 of the 55 studies (<2%) conducted by United Nations (UN) agencies reported gaining ethical approval.^1^ The absence of reporting of ethical oversight of course does not mean that ethical oversight was not obtained. However, our experiences of undertaking research collaboratively with UN agencies, and conversations with colleagues in the field of global health, have led us to the belief that the UN frequently sponsors or participates in studies with human participants that have not received any ethical oversight whatsoever, let alone an appropriate level of ethical oversight for the research project in question.

Explanations for not pursuing ethical oversight provided to us by UN employees have included insufficient time and the belief that the obligation to obtain ethical approval applies to academic researchers alone. In response to one of the author’s requests for comments, one UN agency expressed that they were ‘surprised’ but ‘impressed’ that ethical approval was being sought for a collaborative study with human participants involving the UN agency, raising the possibility that, for some UN agencies, ethical approval is the exception rather than the norm. Concerns have also been raised about the completeness of ethical oversight in UN projects that have received ethical approval. In 2020, the WHO was accused of a ‘serious breach in international ethical standards’ by waiving the requirement of informed consent in the piloting of its malaria vaccination study involving 720 000 children.^2^ Concerns were raised that the WHO had withheld plans to side-step informed consent from the agency’s research ethics committee (REC), with one commentator remarking ‘it is difficult to see how a research ethics committee could have approved a waiver of consent for the WHO malaria vaccine pilot’.^2^ In a case analysis of the malaria vaccine study, van der Graaf *et al* identified a lack of local research ethics approval in addition to insufficient oversight by WHO’s REC and called for greater transparency in WHO’s reporting of the vaccine implementation process.^3^ These examples, combined with our own experiences, have led us to advocate for closer scrutiny of the extent to which UN bodies are conducting and receiving ethical oversight of research with human participants.

Incomplete or insufficient ethical oversight does not appear to be issues confined to the UN. One of the authors of this paper advised a governmental aid agency to establish formal processes for ethical oversight of research and monitoring and evaluation taking place overseas. However, this was seen as an additional burden on an already overstretched workforce, instead opting for the development of ethics guidelines and a reporting system for ethical issues, both of which rely on the will of researchers, consultants and subcontractors to self-police. This article focuses specifically on UN institutions, however, given our particular experience. To our knowledge, there have not been any comprehensive independent reviews undertaken to explore a range of institutional mechanisms supporting the ethical oversight of human participant research conducted by UN bodies. In this paper, we present the findings of a narrative literature review exploring the institutional mechanisms supporting the ethical oversight of human participant research undertaken by UN bodies and discuss whether these mechanisms provide sufficient protection for research participants and their communities.

### Institutional mechanisms supporting ethical oversight of UN research

#### Central level

We identify several possible candidates that could act as designated bodies responsible for the governance of ethical oversight at a central level. One such body is the UN Office for Internal Oversight Services (OIOS), which is responsible for enhancing accountability and transparency within the UN system through its four functions, namely monitoring, internal audit, inspection and evaluation, and investigation.^4^ However, governance of ethical oversight of research appears to lie outside the scope of OIOS’s primarily programme-focused functions. The OIOS manuals guiding each of these functions do not mention ethical oversight of research as part of its core remit.^5^ Another candidate is the UN Ethics Office, the UN body responsible for promoting integrity, professionalism and respect for diversity within the UN. While it is empowered to offer guidance, it does not have governance or investigative authority. In addition, its guidance on staff conduct focuses on issues such as financial transparency and relationships among staff, but it does not appear to cover research conduct.^6^ It is notable that the Staff Regulations and Rules of the United Nations also do not mention oversight of the ethical conduct of research.^7^ A third candidate is the Joint Inspection Unit of the UN, an independent oversight body responsible for conducting UN system-wide evaluations, inspections and investigations; while they have a mandate to cover cross-cutting issues that could include governance of ethical oversight of research activities, to our knowledge, they have not yet undertaken work to this end, although including this responsibility in their scope together with capacity building could make this possible. Central-level bodies would be arguably poorly suited to undertaking ethics review themselves as they are too far removed from research practices, they may lack understanding of the particularities of research under specific UN bodies, and it would not be possible for a central-level body to attain sufficient breadth and depth of expertise to proficiently appraise research proposals in all subject areas under the diverse remits of all UN bodies.

#### Self-governed level

At the level of the UN’s self-governed agencies, funds and other bodies, there is considerable variability in the extent to which institutional mechanisms exist to prevent research with human participants from occurring without ethical oversight. The WHO and UNICEF have dedicated RECs available for research involving these agencies. The WHO and UNICEF also have research ethics governance and accountability processes and quality assurance procedures^8 9^ and dedicated senior staff responsible for governance of research ethics.

However, UNICEF’s Procedure on Ethical Standards in Research, Evaluation, Data Collection and Analysis^9^ deviates from the Declaration of Helsinki^10^ and the UN-generated CIOMS guidelines^11^ by limiting the requirement of independent ethical oversight to research involving vulnerable participants alone rather than to research involving all human participants, leaving it up to researchers themselves to decide whether their proposal meets the arguably ambiguous criteria that UNICEF provides for ascertaining whether a study includes vulnerable participants. Correspondence with UNICEF confirmed this procedure. Researchers cannot be reliably expected to make this determination due to a conflict of interests. In addition, the definition of vulnerability has been contested in the literature^12^,^15^ and it has been argued that vulnerability should not be considered binary in research ethics but on a spectrum of seriousness.^16^ It has also been argued that all human participants should be considered vulnerable given the existence of a power imbalance between the researcher and the participants,^16^ particularly the case if the participants are children. In their procedure, UNICEF identifies the risk of ‘managing expectations and push back from funders and stakeholders consequent to the time lag from additional processes required for appropriate ethical review,’ adding that the mitigation measure is that ‘only sensitive subjects, vulnerable cohorts or risky contexts require external ethical review’.^9^ This honest reporting of the influence of pressure from external actors on research ethics processes provided by UNICEF reflects a commitment to open and transparent reporting. The explicit reporting of this approach in a procedure for ethical standards in research, however, is suggestive of a lack of insight into the ethical unacceptability of having one’s approach to the protection of research participants be determined, in part, by pressure from donors and other external actors. Once again, a conflict of interest argument can be made here. This position casts doubt on the ethical rigour of internal ethical review for those research projects that, according to UNICEF’s procedure, do not require external ethical review.

The institutional mechanisms for preventing research with human participants from occurring without ethical oversight in other UN bodies appear to be almost non-existent or of limited visibility. Ataullahjan *et al* conducted a literature review investigating the existence of research ethics guidelines in UN agencies active in conflict settings.^17^ The review only identified three UN agencies active in conflict settings as having research ethics guidelines, namely the UN Office for the Coordination of Humanitarian Affairs, WHO and UNICEF from nine UN agencies meeting the review’s inclusion criteria.^17^ Research ethics guidelines were not identified from the Food and Agriculture Organization, United Nations Development Programme (UNDP), International Organization for Migration (IOM), United Nations Relief and Works Agency (UNRWA), United Nations High Commissioner for Refugees (UNHCR) or the World Food Programme (WFP). It should be noted that UNRWA has a data protection and disclosure policy that mentions research participants and the agency is currently developing a research ethics policy.^18^ IOM also has a data protection policy providing researchers with a soft prescription that ‘it may be useful to appoint designated persons to oversee research proposals and ensure conformity with any relevant ethical standards associated with various IOM activities’.^19^ IOM also published a research manual in 2004 that provides guidance on ethical principles, although unfortunately this could not be located, possibly accounting for the lack of IOM’s inclusion in Ataullahjan *et al*’s list of UN agencies with research ethics guidelines. UNHCR has coauthored a policy brief entitled Conducting Rigorous Research in Humanitarian Contexts, although its guidance on ethical oversight is limited to a simple explanation that REC oversight may be needed for research conducted in crisis settings.^20^ WFP also has a data protection and privacy policy that provides guidance on deidentification of participants, although it does not mention independent ethical oversight.^21^ It cannot be assumed that employees will fully read the information in research ethics guidelines given the substantial number of documents presented to UN staff, even if the guidelines do align with international ethical standards.

The large majority of UN bodies do not have dedicated RECs, even though many, if not most, sponsor or participate in research with human participants. While some UN bodies have governance and accountability processes and quality assurance procedures, they typically do not cover research activities. Some UN bodies offer online training materials that may include reference to ethical oversight of research. However, the evidence for mandatory training modules as an effective teaching tool has previously been questioned.^22^

### Why should the UN pursue a high standard of ethical oversight of its research with human participants?

Given the important research being undertaken by the UN, one might adopt the position that pursuing increased ethical oversight is a misguided, pedantic pursuit that could jeopardise the feasibility of valuable research projects through the introduction of additional, unwelcome bureaucracy. Yet, there are important justifications for this pursuit.

#### Protection of research participants

The most obvious argument for the ethical oversight of research with human participants involving UN institutions is the identification of research activities that present a risk to participants/communities, affording the opportunity for alternative solutions to be proposed. As mentioned previously, by virtue of the nature of its work, UN research commonly involves participants who are vulnerable for various reasons, and these projects are frequently led by researchers who are relatively unfamiliar with local contexts. UN research projects are often collaborative and complicated by language, cultural, religious, legislative and administrative challenges, furthering the argument for independent oversight to ensure that the interests of all stakeholders are upheld. These complicating factors appeal to the involvement of local researchers and ethical review in the host country.

#### Protection from reputational damage

Second, the reputation of the UN system might be harmed if they are perceived as compromising on research integrity. A lack of internal coherence, or perhaps hypocrisy, may be levelled at the UN which has pioneered and endorsed international standards on research ethics, which notably include the requirement of ethical oversight for all research with human participants.^10 23^ In 2001, UNDP/World Bank/WHO developed the Strategic Initiative for Developing Capacity in Ethical Review (SIDCER), an initiative designed to build regional structures and activities around the globe to better protect research participants. This initiative is grounded in the vision that ‘every research project should be required to undergo proper scientific and ethical review’.^24^ While it could be argued that this standard also applies to the UN, SIDCER was created with an external focus on developing the research ethics capacities of its member countries.^24^

In the case of researchers from high-income countries undertaking research in low-ncome and middle-income countries without ethical oversight, such activities might be perceived as emblematic of neocolonialism, exploitation, the commodification of data or even racism. This can potentially exacerbate the scepticism of, or even distrust in, the UN that has been fuelled by repeated misconduct allegations, such as reports of sexual violence by UN peacekeeping forces.^25^ The threat of reputational damage is arguably greater given that concerns have previously been raised regarding the extent to which the UN is accountable for its actions.^26 27^ In addition, given that many countries have laws requiring ethical oversight for research with human participants, violating these laws could threaten relations between the UN and host government institutions.

#### Ethical standard setting

Third, given the UN’s standard-setting mandate, with a diverse and global set of actors following its lead, failure to achieve its own benchmarks might disincentivise other actors from pursuing ethical oversight and engender a norm that ethical oversight is an aspirational ideal, rather than an essential obligation that safeguards human rights and dignity.

Consistency is imperative; even if a particular research activity presents minimal risk to participants/communities, forgoing ethical oversight without due process on the basis of assumed low risks may contribute to normalising a complacent attitude towards ethical conduct and delegate ethical risk assessments to individual judgement. In addition to assuming a level of ethics expertise that cannot necessarily be taken for granted, as aforementioned, this approach is vulnerable to an inevitable conflict of interests. This could lead to a slippery road towards sloppy scrutiny of other studies, some of which may pose a higher risk and have more harmful consequences. Our position may be articulated as rule utilitarianism, namely the adherence to a moral rule (in this case, ethical oversight) that is deemed to achieve the greatest utility (in this case, the protection of participants and the improvement of the research process as a whole). We argue that if the UN is to remain consistent with its mandate of standard setting, it must carefully balance the need for pragmatism that leads utilitarian considerations on a case-by-case basis (act utilitarianism), with the need to uphold the highest ethical standards. Fast-tracking, let alone forgoing, the ethical review of research perceived as posing minimal risk to participants should be the exception and not the rule.^28^

This said it is important to clarify that we are not advocating for a box-ticking approach to ethical oversight that relies on rigid adherence to process. Ethical scrutiny of research should not only serve to safeguard the interests of research participants but should also help enhance researchers’ reflexivity about their research practices (methodologies, questions, theoretical paradigms, etc). Ultimately, ethical scrutiny should be seen as a key stage of the research process, and a critical space for self-reflection that enhances the quality of research.

#### Enhancing the value of research

Fourth, and linked to the point above, ethical review provides a valuable opportunity for constructive feedback on the research proposal. Duplication of research and inappropriate selection of methods are common in research with human participants globally, not only risking the generation of research that is of limited value underpinned by inefficient use of resources but potentially contributing to the generation of ineffective or harmful interventions and misdirecting further research. Ethical oversight is perhaps the most efficient means of ensuring that some independent scientific review of research proposals is undertaken, although it should not be used as a surrogate for independent scientific peer-review.

## Conclusion

The institutional mechanisms in place to prevent research with human participants from being undertaken by the UN without ethical oversight do not appear, at present, to extend substantively beyond the provision of guidelines and online training, offered by a minority of UN bodies at the self-governed level alone. The WHO and UNICEF are notable exceptions, having implemented various measures to prevent health research with human participants from being undertaken without ethical oversight. However, the WHO and UNICEF are not the only UN bodies that undertake health research with human participants and there are countless actors under the umbrella of the UN system that are regularly involved in non-health research with human participants. Both health and non-health research can present risks to participants and their communities. We acknowledge the need for rigorous empirical research to map out the actual extent of ethical oversight of research conducted by UN bodies, as well as the institutional cultures and processes that help or hinder ethical reflection and the pursuit of ethical oversight. This would support the development of concrete and feasible recommendations. As a starting point, and to further explore concerns of deviation from international standards, the UN could consider the creation of an interagency task force to rigorously and transparently interrogate its institutional mechanisms supporting ethical oversight of research. The suspension of research activities undertaken by UN institutions, that limit external research ethics review to some, but not all, vulnerable participants, may be needed to provide sufficient space for institutional processes and guidelines to be revised in dialogue with independent experts to align with international research ethics standards.

We advocate for a central directive mandating that all UN bodies that sponsor or participate in research with human participants have dedicated research governance and accountability procedures supporting ethical oversight and incorporate the requirement of due process vis-à-vis ethical oversight in all research tendering processes. A lack of in-house capacity for ethical review could be addressed by institutional arrangements with universities and other research organisations to provide ethics review, bypassing the need to create novel structures. External ethics review, however, would still require designated staff within each body to ensure that ethical oversight is performed to the required standards, in addition to financial investments to compensate institutions offering ethics review services. This approach would, however, ensure greater independence of oversight. Historically, the UN has served a valuable role in setting the standards for ethical oversight of research with human participants globally. Is it now time for the UN to set the standards for the oversight of ethical oversight?

## Data Availability

No data are available.
